# Water opens the door to organolithiums and Grignard reagents: exploring and comparing the reactivity of highly polar organometallic compounds in unconventional reaction media towards the synthesis of tetrahydrofurans†
†Dedicated to Professor Paul Knochel on the occasion of his 60^th^ birthday.
‡
‡Electronic supplementary information (ESI) available: Experimental procedures, spectroscopic data of compounds **2a**, **2c**, **3a–n**, and **4a**, and copies of ^1^H and ^13^C NMR spectra of compounds **2a**, **2c**, **3h**, **3i**, and **3m**. See DOI: 10.1039/c5sc03436a


**DOI:** 10.1039/c5sc03436a

**Published:** 2015-11-03

**Authors:** Luciana Cicco, Stefania Sblendorio, Rosmara Mansueto, Filippo M. Perna, Antonio Salomone, Saverio Florio, Vito Capriati

**Affiliations:** a Dipartimento di Farmacia-Scienze del Farmaco , Università di Bari “Aldo Moro” , Consorzio C.I.N.M.P.I.S. , Via E. Orabona 4, I-70125 , Bari , Italy . Email: vito.capriati@uniba.it

## Abstract

It has always been a firm conviction of the scientific community that the employment of both anhydrous conditions and water-free reaction media is required for the successful handling of organometallic compounds with highly polarised metal–carbon bonds. Herein, we describe how, under heterogeneous conditions, Grignard and organolithium reagents can smoothly undergo nucleophilic additions to γ-chloroketones, on the way to 2,2-disubstituted tetrahydrofurans, “on water”, competitively with protonolysis, under batch conditions, at room temperature and under air. The reactivity of the above organometallic reagents has also been investigated in conventional anhydrous organic solvents and in bio-based eutectic and low melting mixtures for comparison. The scope and limitations of this kind of reaction are discussed.

## Abstract 2

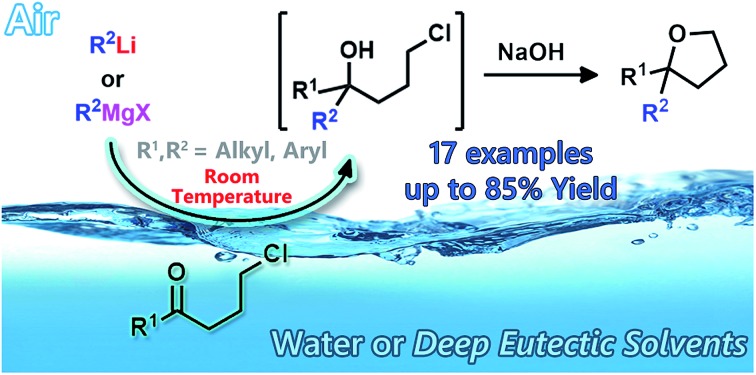
Grignard and organolithium reagents undergo smooth nucleophilic additions to γ-chloroketones “on water”.

## Introduction

For more than one hundred years since their discovery, the life of organometallic compounds of s-block elements has been crippled by the manacles of segregation in an inert atmosphere, and generations of organic chemists have been trained to handle them under rigorously anhydrous conditions. Isn't there any hope of routinely carrying out reactions of highly polar organometallic reagents in aqueous/protic media?^1^

In the last few years, the environmental impact of chemical processes has posed severe and compelling demands for sustainable chemistry, and the development of cost-effective and environmentally benign reaction systems, especially in drug product manufacturing, has become one of the main topics of modern synthetic chemistry.^2^ Green technologies actively look for new solvents to replace conventional harsh organic solvents that present inherent toxicity and high volatility.^3^ The field of aqueous organic synthesis, in particular, is rapidly growing, engaging and attracting, and excellent papers/reviews are being continuously written year in, year out.^4^ This is because water is the prototypical green solvent, being abundant, cheap, non-toxic for living organisms, non-flammable, and with unique physical and chemical properties such as, for example, a large heat capacity, thereby allowing exothermic processes to be operated safer and at room temperature (RT). Moreover, reactions of water-insoluble substrates usually lead to the formation of water-insoluble products whose isolation can be easily carried out by conventional filtration (in the case of solids) or by phase separation (in the case of liquids).

Organometallic chemistry has become a cornerstone of modern organic synthesis, and in recent years there has been growing interest towards aqueous organometallic reactions over those taking place in conventional organic solvents.1*b* However, although water is increasingly being used (both mixed with organic solvents and in bulk) in the chemistry of d-and p-block elements,1b,^5^ its employment in the chemistry of s-block elements (mainly organolithiums and Grignard reagents) is still limited to catalytic or stoichiometric amounts with surprisingly beneficial effects on reaction rate, product yield, and regio- and stereochemistry.1b,^6^

Interestingly, Barbier–Grignard-type reactions run in water are also taking to the stage today. The first magnesium-mediated Barbier–Grignard allylation of aldehydes in water was reported in 1998 by Li and Zhang.7*a* Such reactions also succeeded when performing the direct carbonyl alkynylation, phenylation, alkylation, and arylation using non-activated halides in the presence of various metals (*e.g.* Zn, CuI) and with the assistance of In(i) salts or transition metals such as Rh.7b–f

The potential impact of unconventional reaction media on the chemistry of s-block elements has recently been independently investigated by Hevia, García-Álvarez and co-workers^8^ and by our group^9^ employing the so-called “deep eutectic solvents”, which are fluids generally composed of two or three safe and inexpensive components that can undergo self-association through hydrogen-bond interactions, thereby forming an eutectic mixture with a melting point lower than either of the individual components and with unusual solvent properties.^10^ Both nucleophilic additions and substitutions^8^,9a promoted by Grignard and organolithium reagents proved to be effective in such unconventional solvents, thereby providing the expected adducts in good yields and competitively with protonolysis. Novel organometallic transformations have been also successfully explored and carried out directly in a glycerol-containing bio-based mixture.9*b* In a recent paper, Madsen and Holm showed that once solutions of highly reactive Grignard reagents (allylmagnesium bromide or benzylmagnesium chloride) and of substrates (acetone or benzaldehyde) were prepared separately in syringes and pressed against one another by means of polyethylene capillary tubes in the presence of water, the rate of carbonyl addition efficiently competed with that of protonation.^11^ Such an intermolecular competition in flow, however, failed in the case of the less reactive alkyl Grignard reagents. We herein investigate for the first time the potential benefits of using a heterogeneous solvent mixture in the nucleophilic addition of both Grignard and organolithium reagents to carbonyl derivatives “on water”, under air, at room temperature and in batch conditions.

## Results and discussion

### A. Reactions in anhydrous THF

To tackle this problem, we initiated our study by using 4-chloro-1-phenylbutan-1-one (**1a**) as a model substrate for the preparation of 2,2-disubstituted tetrahydrofuran **3a**^12^*via* intramolecular cyclization of the intermediate chlorohydrin **2a** upon reaction with commercially available MeLi (1.6 M Et_2_O solution) or MeMgCl (3.0 M THF solution) in anhydrous THF and under a nitrogen atmosphere for comparison (Table 1). The preparation of this substrate from an enolizable ketone also offers the possibility to gain more information about the less/more pronounced nucleophilic/basic character exhibited by the employed organometallic reagent, under certain experimental conditions, by monitoring the competitive formation of other potentially attainable products.

**Table 1:** Addition reaction of MeMgCl and MeLi (RM) to γ-chloroketone **1a** in anhydrous THF

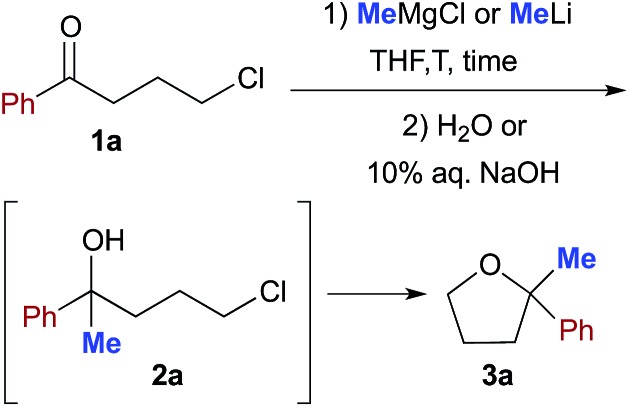						
Entry	RM (equiv.)	*T* (°C)	Time	**1a** yield%	**2a** yield%	**3a** yield%
1	MeMgCl (3)^*a*^	–40	10 min	72^*b*^	25^*b*^	3^*b*^
2	MeMgCl (3)^*a*^	–40^*c*^	12 h	20^*b*^	35^*b*^	35^*b*^
3	MeMgCl (3)^*d*^	–40	12 h	—	—	60^*e*^
4	MeMgCl (6)^*d*^	–40	12 h	—	—	80^*e*^
5	MeMgCl (3)^*d*^	RT	12 h	—	—	10^*b*^ ^,^^*f*^
6	MeMgCl (3)^*d*^ ^,^^*g*^	–40	12 h	—	—	<5^*b*^ ^,^^*f*^ ^,^^*h*^
7	MeLi (3)^*a*^	–40	10 min	40^*b*^	60^*b*^	—
8	MeLi (3)^*a*^	–40^*c*^	12 h	—	38^*b*^	38^*b*^
9	MeLi (3)^*d*^	–40	12 h	—	—	70^*e*^
10	MeLi (6)^*d*^	–40	12 h	—	—	85^*e*^
11	MeLi (3)^*d*^	RT	12 h	—	—	30^*e*^ ^,^^*f*^

^*a*^Upon quenching with H_2_O.

^*b*^Determined by ^1^H NMR analysis of the crude reaction mixture.

^*c*^From –40 °C to RT.

^*d*^Upon treatment with 10% aq. NaOH, 3 h.

^*e*^Isolated yield after column chromatography.

^*f*^A mixture of unidentified products also formed.

^*g*^Neat conditions.

^*h*^Same result at –40 °C.

When a THF (1 mL) solution of **1a** (0.5 mmol) was reacted with MeMgCl (3 equiv.) or MeLi (3 equiv.) at –40 °C, and quenched after 10 min reaction time with H_2_O, mainly a mixture of unreacted substrate (up to 72% in the reaction with MeMgCl) and chlorohydrin **2a** (up to 60% in the reaction with MeLi) was detected in the crude product (Table 1, entries 1 and 7). The spontaneous intramolecular cyclization of **2a** to **3a** in the presence of the organometallic reagent, however, proved to be slow in THF because a 1 : 1 mixture of **2a** and **3a** was still present after 12 h stirring at RT (Table 1, entries 2 and 8). Upon further treatment with 10% aq. NaOH (3 h), THF derivative **3a** could finally be isolated in 60 and 70% yields in the reactions with MeMgCl and MeLi, respectively (Table 1, entries 3 and 9). By increasing the amount of the organometallic reagent to up to 6 equiv., the corresponding yields of **3a** were found to increase to up to 85% (Table 1, entries 4 and 10). Conversely, upon running the reaction at RT, the yield of **3a** considerably decreased to 10–30% (Table 1, entries 5 and 11), whereas only a trace of product (<5%) was detected both at RT and at –40 °C under neat conditions (Table 1, entry 6).

### B. Reactions in deep eutectic solvents and low melting mixtures

**Fig. 1:**
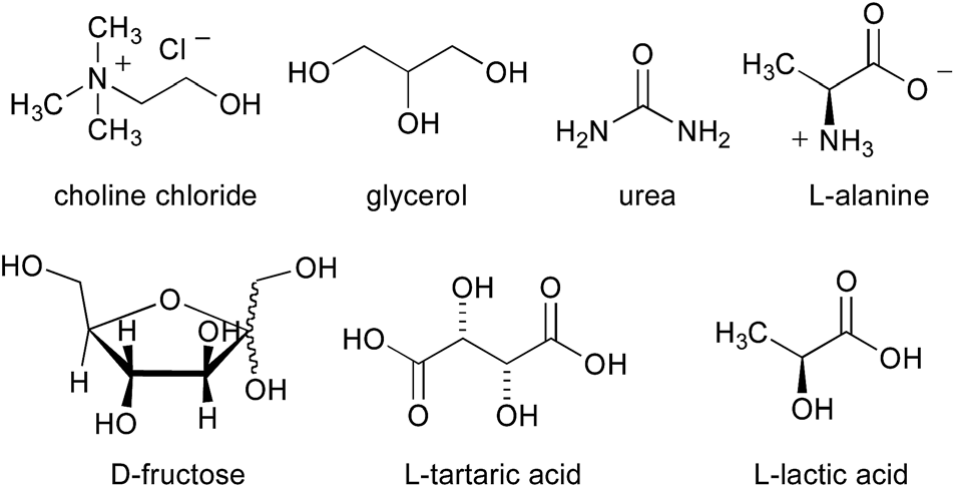
Components of DES/low melting mixtures used in the present study.

The next investigation was to study the effect of different bio-based deep eutectic solvents (DESs) and low melting mixtures (LMMs)^13^ based on carbohydrates/urea (Fig. 1) on the chemoselectivity of the addition reaction of the aforementioned organometallic reagents to γ-chloroketone **1a**. The addition of a solution of MeMgCl (3 equiv.) to **1a** (0.5 mmol) in a d-fructose–choline chloride (ChCl) (2 : 1) eutectic mixture, at RT and under air, gave 66% conversion to **2a** and 18% conversion to **3a** after just 10 min reaction time (Table 2, entry 1). It should be noted that a higher conversion was detected both in a d-fructose–urea LMM (3 : 2) (up to 81% of **2a**) (Table 2, entry 3) and in a ChCl–glycerol (Gly) (1 : 2) DES mixture (up to 85% of **2a**) (Table 2, entry 5). On the other hand, if the acidity of the reaction medium increases considerably, as with the employment of l-tartaric acid–ChCl (1 : 2) and l-lactic acid–l-alanine (9 : 1) eutectic mixtures, essentially complete protonation of the Grignard reagent is observed with substantial or quantitative recovery of the unreacted ketone (Table 2, entries 7 and 8).

**Table 2:** Addition reaction of MeMgCl and MeLi (RM) to γ-chloroketone **1a** in DES/low melting mixtures

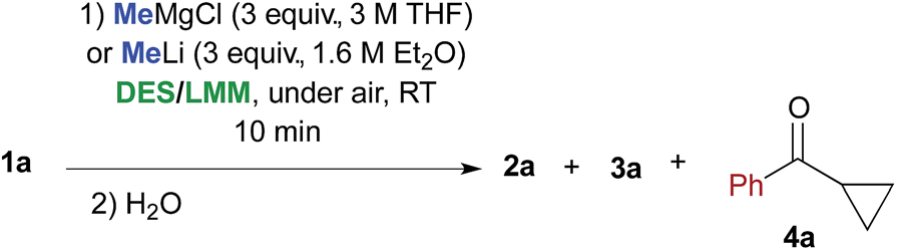						
Entry	RM	DES/LMM^*a*^	**1a** yield^*b*^%	**2a** yield^*b*^%	**3a** yield^*b*^%	**4a** yield^*b*^%
1	MeMgCl	DES **A**	16	66	18	—
2	MeLi	DES **A**	12	—	28	55
3	MeMgCl	LMM **A**^*c*^	19	81	—	—
4	MeLi	LMM **A**^*c*^	15	26	26	33
5	MeMgCl	DES **B**	15	85	—	—
6	MeLi	DES **B**	10	63	10	12
7	MeMgCl	DES **C**^*d*^	70	30	—	—
8	MeMgCl	DES **D**	100	—	—	—

^*a*^1 g per 0.5 mmol of **1a**; DES **A**: d-fructose–ChCl (2 : 1, mol mol^–1^); LMM **A**: d-fructose–urea (3 : 2, w/w); DES **B**: ChCl–Gly (1 : 2, mol mol^–1^); DES **C**: l-tartaric acid–ChCl (1 : 2, mol mol^–1^); DES **D**: l-lactic acid–l-alanine (9 : 1, mol mol^–1^).

^*b*^Determined by ^1^H NMR analysis of the crude reaction mixture.

^*c*^Reaction run at 65 °C.

^*d*^Reaction run at 50 °C.

When performed in the above DES/low melting mixtures using MeLi, such addition reactions proved to be less effective. The remaining mass balance in this reaction is mostly accounted for by the formation of cyclopropyl phenyl ketone **4a** in variable amounts as the main by-product, which probably arises from an intramolecular displacement of the chloride ion by the enolate intermediate (Table 2, entries 2, 4 and 6). These results suggest that in suitable DES media, the basic character of the organolithium reagent is much more pronounced than its nucleophilic character compared with those of the corresponding Grignard reagent, the latter also being able to promote the formation of compounds **2a** and **3a** at a higher conversion rate than in THF.^14^

**Scheme 1:**
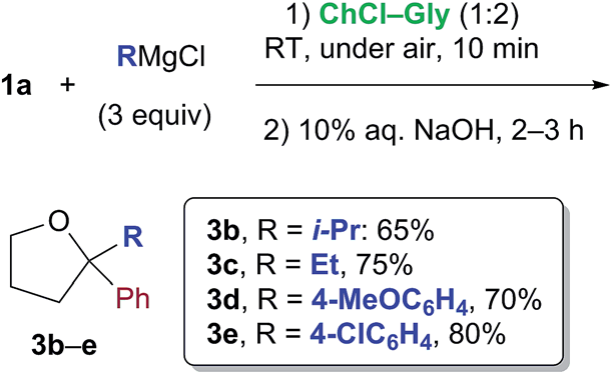
Formation of THF derivatives **3b–e***via* nucleophilic addition of Grignard reagents to γ-chloroketone **1a** in a ChCl-based eutectic mixture at RT and under air.

Cognizant of the above achievements, we were pleased to find that the nucleophilic addition of other Grignard reagents (*i*-PrMgCl, EtMgCl, 4-MeOC_6_H_4_MgBr and 4-ClC_6_H_4_MgBr) to the above enolizable ketone (**1a**), run in the ChCl–Gly (1 : 2) eutectic mixture, and followed by treatment with NaOH, straightforwardly furnished the expected 2,2-disubstituted tetrahydrofurans **3b–e** in 65–80% yield under air and at RT (Scheme 1). These results are thus in agreement with previous findings by Hevia and García-Álvarez.^8^ It is worth noting, however, that the employment of carbohydrate urea melts (*e.g.* LMM **A**, Table 2) can also be useful for successfully carrying out the above nucleophilic additions (Table 2, entry 3).

### C. Reactions on water

Can we replace a bio-based eutectic mixture with water? A preliminary experiment showed that when 1 equiv. of MeMgCl (3.0 M THF solution) or MeLi (1.6 M Et_2_O solution) was rapidly spread over a suspension of **1a** (0.5 mmol) in water (1 mL) at RT and under air, a very poor conversion of starting material into chlorohydrin **2a** resulted after 10 min (up to 20% yield, Table 3, entries 1 and 2). By employing 2 equiv. of either MeMgBr or MeLi, chlorohydrin **2a** was detected in up to 50% yield in the crude reaction mixture (Table 3, entries 3 and 4). Pleasingly, upon switching to 3 equiv., γ-chloroketone **1a** underwent nucleophilic addition by MeMgBr and MeLi affording chlorohydrin **2a** in 70 and 72% yields, respectively (Table 3, entries 5 and 9). It should be noted that the corresponding percentage conversions in anhydrous THF at –40 °C after 10 min were only 25% (MeMgCl) and 60% (MeLi) (compare with Table 1, entries 1 and 7). Treatment of the crude reaction mixtures with 10% aq. NaOH finally led to the direct isolation of THF derivative **3a** in up to 75% yield (Table 3, entries 6 and 10). This latter value could be further improved to up to 82% by using 6 equiv. of the above organometallic reagents (Table 3, entries 7 and 11). The employment of a larger volume of water (3 mL), however, produced a considerable decrease in the yield of **3a** from 75% to 45% further to the addition of MeLi (3 equiv.) (Table 3, entry 12).

**Table 3:** Addition reaction of MeMgCl and MeLi (RM) to γ-chloroketone **1a** “on water”

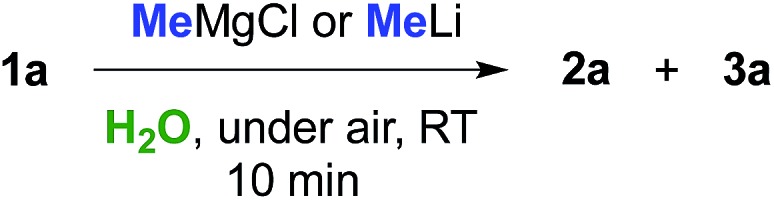				
Entry	RM (equiv.)	**1a** yield%	**2a** yield%	**3a** yield%
1	MeMgCl (1)	82^*a*^	18^*a*^	—
2	MeLi (1)	80^*a*^	20^*a*^	—
3	MeMgCl (2)	71^*a*^	29^*a*^	—
4	MeLi (2)	50^*a*^	50^*a*^	—
5	MeMgCl (3)	20^*a*^	70^*a*^	5^*a*^
6	MeMgCl (3)^*b*^	—	—	72^*c*^ ^,^^*d*^
7	MeMgCl (6)^*b*^	—	—	80^*c*^ ^,^^*d*^
8	MeMgCl (3)^*b*^	—	—	35^*c*^ ^,^^*d*^ ^,^^*e*^
9	MeLi (3)	18^*a*^	72^*a*^	5^*a*^
10	MeLi (3)^*b*^	—	—	75^*c*^ ^,^^*d*^
11	MeLi (6)^*b*^	—	—	82^*c*^ ^,^^*d*^
12	MeLi (3)^*b*^	—	—	45^*c*^ ^,^^*d*^ ^,^^*f*^

^*a*^Determined by ^1^H NMR analysis of the crude reaction mixture.

^*b*^Upon treatment with 10% aq. NaOH, 3 h.

^*c*^Isolated yield after column chromatography.

^*d*^Ketone **4a** could also be isolated in 15–20% yield.

^*e*^After removing most of the THF under vacuum from a commercial solution of MeMgCl.

^*f*^Water: 3 mL.

With satisfactory conditions found for MeLi and MeMgBr, we sought to capitalize on this by exploring the scope of the reaction with a variety of substrates and organometallic reagents. Assorted aliphatic and aromatic Grignard and organolithium reagents such as *i*-PrMgCl/*i*-PrLi, EtMgCl/EtLi, *n*-BuLi, allylMgCl, *p*-anisylMgBr and *p*-chlorophenylMgBr all proved to be effective in the nucleophilic addition to a suspension of **1a** in water, at RT and under air, straightforwardly providing the expected THF derivatives **3b–g** in satisfactory yields (3 equiv.: 50–75%; 6 equiv.: 58–85%) upon final treatment with 10% aq. NaOH (Table 4).

**Table 4:** Addition reaction of organometallic reagents to γ-chloroketones **1a–d** “on water”, under air and at RT, to afford 2,2-disubstituted tetrahydrofuran derivatives **3a–n**

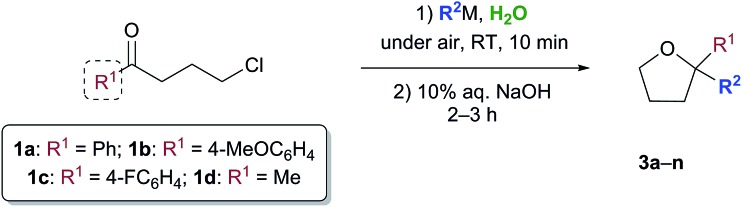
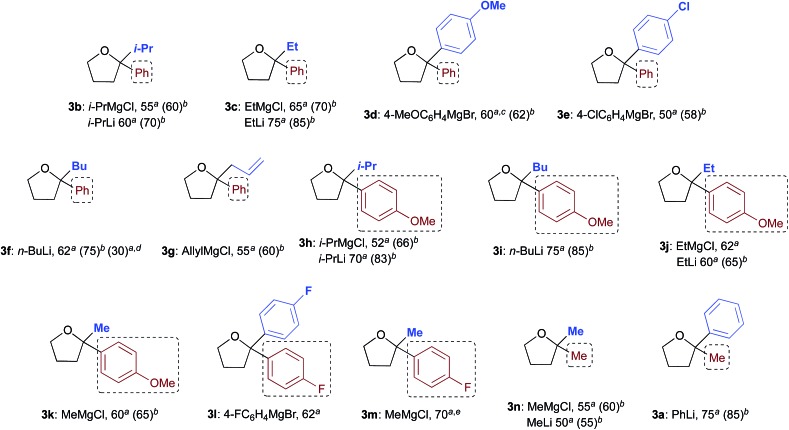

^*a*^3 equiv. of R^2^M (isolated yields).

^*b*^6 equiv. of R^2^M (isolated yields).

^*c*^Compound **3d** could also be obtained (70% yield) by reacting γ-chloroketone **1b** with PhLi (3 equiv.).

^*d*^After removing most of the hexanes under vacuum from a commercial solution of *n*-BuLi.

^*e*^Compound **3m** could also be obtained in 80 and 85% yield by reacting γ-chloroketone **1d** with 3 and 6 equiv. of 4-FC_6_H_4_MgBr, respectively.

Similarly, when an aryl-substituted ketone with an electron-donating group (**1b**) was used as a substrate, adducts **3h–k** were isolated in 52–75% yield with 3 equiv. and in up to 85% yield employing 6 equiv. of RMgCl/RLi (Table 4). The presence of a fluorine atom on the aromatic ring (**1c**) was well tolerated in the addition reactions with both fluorinated and aliphatic Grignard reagents (3 equiv.), thereby affording products **3l** and **3m** in 62 and 70% yields, respectively (Table 4). Finally, it was interesting to observe that both aliphatic and aromatic Grignard and organolithium reagents again worked well in the addition reaction to a suspension of the aliphatic γ-chloroketone **1d** in water, thus leading to the corresponding adducts **3a** and **3n** in 50–75% (3 equiv.) or 55–85% yields (6 equiv.). It should be noted that compounds **3d** and **3m** could also be obtained in 60–85% yield by reacting γ-chloroketones **1b** and **1d** with PhLi (3 equiv.) and 4-FC_6_H_4_MgBr (3 or 6 equiv.), respectively.

In a landmark paper published in 2005, Sharpless and co-workers introduced the concept of “on water” reactions when insoluble organic reactants were able to generate high yields of products with substantial rate acceleration once stirred vigorously in pure water for short periods of time.^15^ An interesting and important aspect of this work, often overlooked in the literature, is that a significant solvent isotope effect was also noticed by the authors: the reaction rate decreased when D_2_O was used in place of water. These reactions are thought to occur at the interface between the immiscible phases. The molecular origin of such a rate acceleration, however, has been a matter of dispute. Recently, Huck and co-workers succeeded in quantifying the “on water” effect by using a biphasic (water/toluene) fluidic approach,^16^ which supported a mechanism involving a possible stabilization of both reactants and transition state by *trans*-phase H-bonding according to the model proposed by Jung and Marcus.^17^ On the other hand, some of the well-studied “in water” effects operating in organic reactions for clear solutions of soluble organic reactants are (a) the Breslow hydrophobic effect,^18^ (b) hydrogen-bonding effects on reactants and transition states, and (c) water polarity effects.^19^

The reactions described in the present paper deal with the quick addition of an ethereal/hydrocarbon solution of the organometallic reagent miscible in varying proportions [from completely miscible (*e.g.* THF) to completely immiscible (*e.g.* hexane)] in the water medium to a suspension of the sparingly soluble γ-chloroketone (*ca.* 10^–3^ mol L^–1^)^20^ at RT, under air, and under vigorous stirring. Thus, they are unique *per se* in the scenario of organic transformations in aqueous media investigated so far because the observed chemoselective s-block-metal-mediated nucleophilic additions to the carbonyl derivatives are at the same time in competition with protonolysis processes. Some remarks are in order. By comparing the results of Tables 1 and 3, it transpires that, upon switching from THF to water, comparable yields in **2a** and **3a** can be obtained in shorter reaction times. For example, in the addition reaction of MeMgCl (3 equiv.) to **1a** in dry THF, the amount of starting ketone can be reduced to 20% only after 12 h stirring at RT (overall yield in **2a** and **3a**: 70%) (Table 1, entry 2). Conversely, the same reaction performed “on water” needs only 10 min stirring at RT to produce a similar result and to afford **2a** and **3a** in an overall yield of 75% (Table 3, entry 5).

We also investigated the influence of the ethereal/hydrocarbon solution of the organometallic reagent on the final yield. After removing most of the THF or hexanes under vacuum from a commercial solution containing 3 equiv. of MeMgCl or *n*-BuLi and transferring each of the resulting, very reactive, concentrated solutions to a flask containing **1a**, the expected THF derivatives **3a** and **3f** again formed after treatment with 10% NaOH, albeit in diminished yields (**3a**: 35%; **3f**: 30%) (Table 3, entry 8 and Table 4). Therefore, solvation and dilution of the organometallic reagent is important for better yields.

Does the reaction take place within the organic solvent or at the interface with water? As was pointed out earlier, while organolithium compounds are generally prepared and sold in hydrocarbon solvents which are immiscible with water, most of the Grignard reagents employed in the present study are in a THF solution, which is totally miscible with water. Thus, at least for the latter case, nucleophilic addition should occur at the oil–water phase boundary, with the starting ketone being the only insoluble organic component in the aqueous medium.

**Scheme 2:**
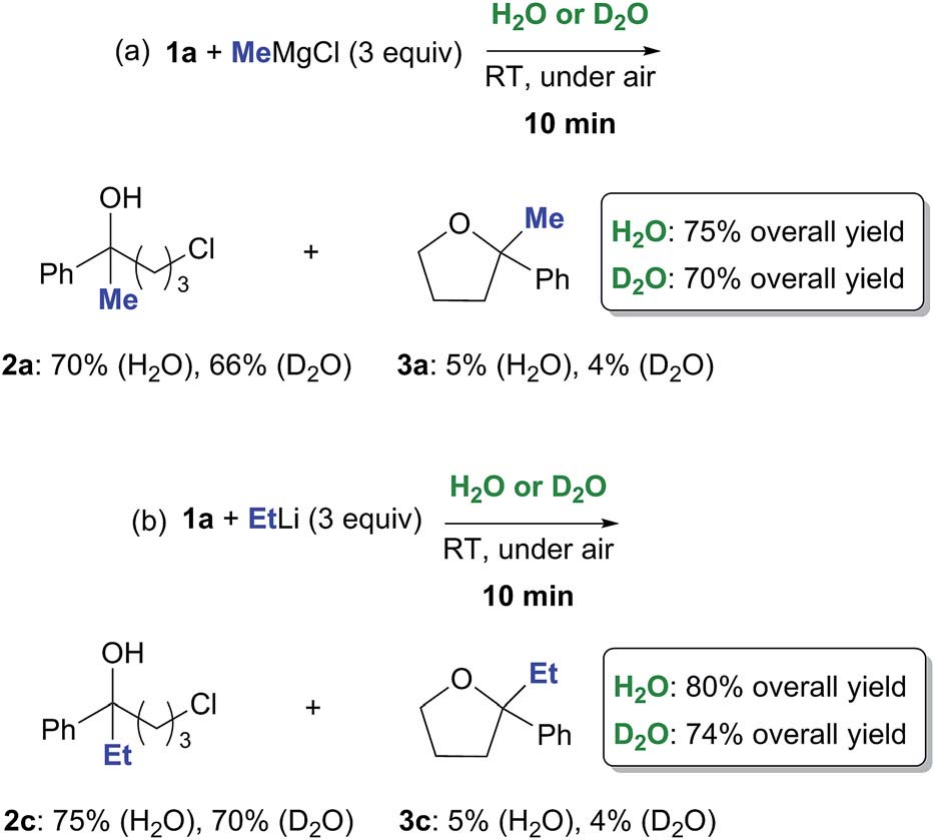
Nucleophilic addition of MeMgCl (a) and EtLi (b) to γ-chloroketone **1a** on H_2_O or on D_2_O at RT and under air.

May the *trans*-phase H-bonding be playing a role in this context? Two sets of reactions were run in parallel on **1a** (0.5 mmol) with MeMgCl (3 equiv., 3.0 M in THF) and EtLi (3 equiv., 0.5 M in benzene/cyclohexane) employing either H_2_O (1 mL) or D_2_O (1 mL) as the aqueous phase (Scheme 2). After 10 min reaction time, each reaction was worked up. The ^1^H NMR analysis of the crude reaction mixtures revealed the presence of chlorohydrins **2a** and **2c** (66–75% yield) and very small amounts (up to 5% yield) of the corresponding THF derivatives **3a** and **3c** as the only products, with the rest being identified as the starting ketone.^21^ Interestingly, a small deuterium kinetic isotope effect (KIE) was observed. Both the reactions run on D_2_O proved, indeed, to be slightly slowed down, the overall yields in the final adducts **2a**/**3a** and **2c**/**3c** decreasing to up to about 8% (Scheme 2).

In contrast to the classic KIE, these kinds of isotope effects, which are not related to the breaking of any chemical bond of water, have been neither fully understood nor closely investigated at aqueous interfaces. A possible explanation focusing on physical factors was brought forward by Marcus and Jung: the higher viscosity of D_2_O may affect the droplet size of the reactants, and thus reaction times.^17^

Pool, Nagata and coworkers, using combined theoretical and experimental approaches, recently demonstrated that the molecular organization and superstructure of water was modified when replacing water (H_2_O) by heavy water (D_2_O).^22^ It was shown, in particular, that the bond orientation of water at the water–vapour interface (which may also serve as a useful model system for extended hydrophobic interfaces) depends markedly on the water isotope composition with the O–H bonds tending to orient up into the vapour phase and the O–D bonds preferably pointing down into the bulk water, thereby leading to stronger hydrogen bonds. This interesting finding could provide an alternative explanation for the usually observed decrease of reaction rate in on D_2_O chemistry: if the number of dangling OD groups in D_2_O (free, not D-bonded) available at the interface reduces considerably, the efficiency of the hydrogen-bond catalysis may be affected as well. By comparing organic reactions on H_2_O and on D_2_O, Butler and Coyne have recently shown that “on water” catalysis can indeed range from weak to strong *trans*-phase H-bonding for reactants according to their basicity, a fact that is consistent with a chameleon-type behavior of water at hydrophobic surfaces.^23^

**Scheme 3:**
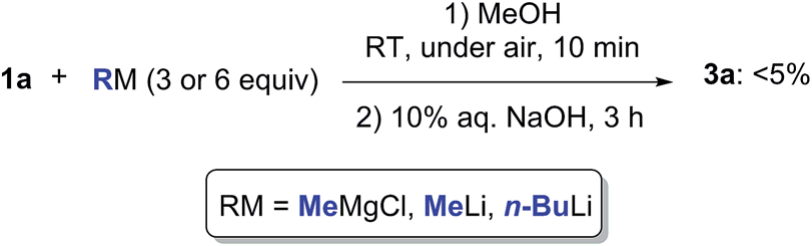
Nucleophilic addition of MeMgCl, MeLi or *n*-BuLi to γ-chloroketone **1a** in MeOH at RT and under air.

Can we replace water with another protic medium? We turned our attention to MeOH in which ketone **1a** proved to be completely soluble. When trying to perform the addition reaction to a solution of **1a** (0.5 mmol) in MeOH (1 mL) with MeMgCl (3 or 6 equiv., 2.0 M in THF), MeLi (3 or 6 equiv., 3% in Et_2_O), or *n*-BuLi (3 or 6 equiv., 2.5 M in hexanes), at RT and under air, a higher degree of protonation was observed, and the expected adduct **3a** could only be detected in traces in the crude reaction mixture (Scheme 3). Thus, the solubility of the reactant is important and may play a role in promoting such nucleophilic additions. The ability of MeOH to engage in hydrogen bonding is also more limited as compared to water.^24^

## Conclusions

Returning to the main issue mentioned at the onset of this research paper, what we have learnt from the present study is : (i) that commercial ethereal/hydrocarbon solutions of organolithium and Grignard reagents (range concentration 0.5–3.0 M), preferably employed in slight excess (at least 3 equiv.) to the amount required to react with the substrate, successfully promote nucleophilic additions once added to a suspension of the enolizable γ-chloroketone in water (0.5 mmol per 1 mL of water). (ii) Both alkylation and arylation of various alkyl and aryl γ-chloroketones take place in satisfactory yields (3 equiv.: up to 75%; 6 equiv.: up to 85% yield) and can be performed under batch conditions, at RT and under air, and competitively with protonolysis. (iii) Critical to the achievement of this advance was the use of heterogeneous conditions (ketones sparingly soluble in water), which are typical of “on water” chemistry. (iv) The solvent isotope effect and the fact that water could not be replaced by alcohols suggest that strong intermolecular hydrogen bonds (and thus the creation of supramolecular clusters in solution) jointly with *trans*-phase H-bonding with the substrate may be playing a key role (a) in shielding the organometallic reagent from competitive protonolysis processes, and (b) in activating the carbonyl derivative towards nucleophilic addition.^25^ (v) Both ChCl-based DESs and low melting mixtures consisting of carbohydrate and urea were found to be similarly effective as alternative reaction media for s-block-metal-mediated nucleophilic carbonyl additions with the difference that the basicity of organolithium reagents proved to be more pronounced compared to that exhibited by Grignard reagents.

Major breakthroughs can be expected in the near future by investigating the structure–reactivity relationships of highly polar organometallic compounds^26^ and by an in-depth understanding of their reaction mechanisms in such unconventional reaction media. It is our hope that these preliminary results will set the stage to encourage the scientific community to deepen the investigations into this amazing but still poorly understood field, so as to unveil novel aspects of reactivity, which will be both intellectually rewarding and of practical significance.

## Supplementary Material

Supplementary information
