# Supplementary material for: Water opens the door to organolithiums and Grignard reagents: exploring and comparing the reactivity of highly polar organometallic compounds in unconventional reaction media towards the synthesis of tetrahydrofurans
Source: Chem Sci. 2015 Nov 3;7(2):1192–9. doi: 10.1039/c5sc03436a (PMC5975938; doi:10.1039/c5sc03436a)

Electronic Supplementary Material (ESI) for Chemical Science.
This journal is © The Royal Society of Chemistry 2015

Electronic Supplementary Information (ESI)

Water opens the door to organolithium and Grignard reagents: exploring and comparing the reactivity of highly polar organometallic compounds in unconventional reaction media towards the synthesis of tetrahydrofurans

Luciana Cicco, Stefania Sblendorio, Rosmara Mansueto, Filippo M. Perna, Antonio Salomone, Saverio Florio, and Vito Capriati*

Dipartimento di Farmacia-Scienze del Farmaco, Università di Bari "Aldo Moro", Consorzio C.I.N.M.P.I.S., Via E. Orabona 4, I-70125, Bari, Italy.

E-mail: vito.capriati@uniba.it

Table of Contents

1. General Methods S2

2. Experimental procedures

   2.1 Preparation of 5-chloro-2-phenylpentan-2-ol (**2a**) and 2-methyl-2-phenyltetrahydrofuran (**3a**) in THF. Typical Procedure. S3

   2.2 Preparation of 2-methyl-2-phenyltetrahydrofuran (**3a**) in deep eutectic solvents. Typical procedure. S4

   2.3 Preparation of 2-methyl-2-phenyltetrahydrofuran (**3a**) in water. Typical procedure. S5

3. Characterization data S6

4. ^{1}H and ^{13}C NMR spectra S11

1. General Methods

Tetrahydrofuran (THF), was freshly distilled, under a nitrogen atmosphere, over sodium/benzophenone ketyl. Eutectic mixtures of solvents [choline chloride (ChCl)−glycerol (Gly) (1:2 mol/mol); D-fructose−ChCl (2:1 mol/mol); L-tartaric acid−ChCl (1:2 mol/mol); L-lactic acid−L-alanine (9:1 mol/mol)] and the low melting mixture of D-fructose−urea (3:2 weight/weight) were prepared by heating under stirring up to 90 °C for 10–30 min the corresponding individual components until a clear solution was obtained. For ^{1}H and ^{13}C NMR spectra (^{1}H NMR 400, 500 or 600 MHz; ^{13}C NMR 100, 125 or 150 MHz), $CDCl_3$ was used as the solvent. GC-MS spectrometry analyses were performed on a gas chromatograph (dimethylsilicon capillary column, 30 m, 0.25 mm i.d.) equipped with a mass selective detector operating at 70 eV (EI). Elemental analyses were performed by using a Carlo Erba CHNS-O EA1108-Elemental Analyzer. Analytical thin layer chromatography (TLC) was carried out on precoated 0.25 mm thick plates of Kieselgel 60 F254; visualization was accomplished by UV light (254 nm) or by spraying with a solution of 5 % (w/v) ammonium molybdate and 0.2 % (w/v) cerium(III) sulfate in 100 ml 17.6 % (w/v) aq. sulfuric acid and heating to 473 K for some time until blue spots appear. Some reactions involving air-sensitive reagents were performed under argon in oven-dried glassware using syringe-septum cap technique. The following solutions of Grignard reagents and organolithium reagents were commercially available and were used with the following concentration: MeMgCl 3.0 M in THF, EtMgCl 2.0 M in THF, *i*-PrMgCl 2.0 M in THF, allylMgCl 2.0 M in THF, 4-$MeOC_6H_4MgBr$ 0.5 M in THF, 4-ClC_6H_4MgBr 1.0 M in 2-MeTHF, 4-FC_6H_4MgBr 2.0 M in Et_2O, MeLi 1.6 M in Et_2O, EtLi 0.5 M in benzene/cyclohexane, *i*-PrLi 0.7 M in pentane, *n*-BuLi 2.5 M in hexanes, PhLi 1.8 M in dibutyl ether. Spectroscopic data of compounds **3a**,[1] **3b**,[2] **3c**,[3] **3d,e**,[4] **3f**,[5] **3g**,[3] **3j**,[6] **3k**,[7] **3l**,[4] **3n**[8] and **4a**[9] are in agreement with the literature. γ−Chloroketones **1a–d** and cyclopropyl phenyl ketone **4a** are commercially available. Fully characterization data, including elemental analysis and copies of ^{1}H and ^{13}C NMR spectra, have been reported for the new compounds **2a**, **3h**, **3i** and **3m**.

---

[1] E. D. Butova, A. V. Barabash, A. A. Petrova, C. M. Kleiner, P. R. Schreiner and A. A. Fokin, *J. Org. Chem.* 2010, **75**, 6229.
[2] G. A. Moniz and J. L. Wood, *J. Am. Chem. Soc.*, 2001, **123**, 5095.
[3] R. Mansueto, V. Mallardo, F. M. Perna, A. Salomone and V. Capriati, *Chem. Commun.* 2013, **49**, 10160.
[4] V. Mallardo, R. Rizzi, F. C. Sassone, R. Mansueto, F. M. Perna, A. Salomone and V. Capriati, *Chem. Commun.* 2014, **50**, 8655.
[5] Y. Maeda, T. Nishimura and S. Uemura, *Chem. Lett.* 2005**, 34**, 790.
[6] C. Zhu and J. R. Falck, *Angew. Chem. Int. Ed.* 2011**, 50***,* 6626.
[7] P. P. Singh, S. Gudup, H. Aruri, U. Singh, S. Ambala, M. Yadav, S. D. Sawant and R. A. Vishwakarma, *Org. Biomol. Chem.*, 2012*,* **10**, 1587.
[8] A. K. Diba, J. Begouin and M. Niggemann, *Tetrahedron Lett.,* 2012*,* **53,** 6629.
[9] C. Clarke, S. Foussat, D. J. Fox, D. S. Pedersen and S. Warren, *Org. Biomol. Chem.,* 2009*,* **7**, 1323.

2. Experimental Procedures

2.1 Preparation of 5-chloro-2-phenylpentan-2-ol (2a) and 2-methyl-2-phenyltetrahydrofuran (3a) in THF. Typical Procedure.

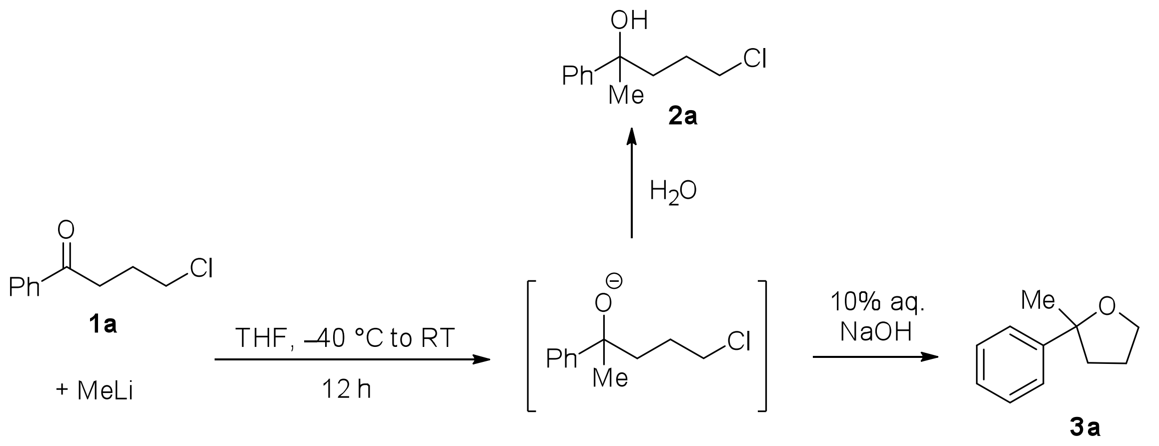

To an anhydrous THF solution (1 mL) of the ketone **1a** (0.5 mmol), 0.94 mL of the commercially available MeLi (1.5 mmol in 1.6 M Et_2O solution) were added dropwise, under argon, at –40 °C. After 12 h stirring at RT, the reaction mixture was treated with 10% aq. NaOH for 3 h (or quenched with H_2O to isolate chlorohydrin **2a**), and then extracted with Et_2O (3 × 20 mL). The combined organic phases were dried over Na_2SO_4 and the solvent was concentrated *in vacuo*. The crude product was purified by flash-chromatography (silica gel, hexane/Et_2O 80:20, Et_3N 2%), to give **3a** in 70% yield. Spectroscopic data are in accord with the literature.[1] The chlorohydrin **2a** could be purified by flash-chromatography on silica gel with hexane/AcOEt 80:20 as the eluent in 38% yield (see Table 1 of the main text).

2.2 Preparation of 2-ethyl-2-phenyltetrahydrofuran (3c) in deep eutectic solvents. Typical procedure.

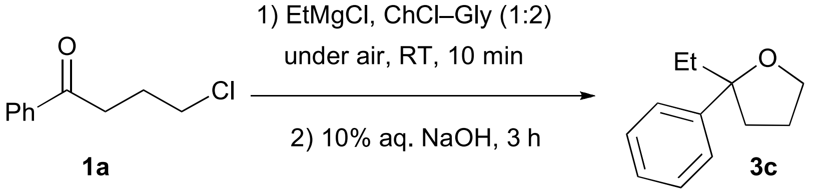

To a mixture of the ketone **1a** (0.5 mmol) in 1 g of ChCl−Gly (1:2), 0.75 mL of the commercially available EtMgCl (1.5 mmol in 2.0 M THF solution), handled under argon using conventional Schlenk techniques, were quickly spread out at RT, under air, and vigorous stirring. After 10 min, 10 mL of 10% aq. NaOH were added and the mixture was stirred for additional 3 h, and then extracted with Et_2O (3 × 10 mL). The combined organic phases were dried over anhydrous Na_2SO_4 and the solvent was concentrated *in vacuo*. The crude product was purified by flash-chromatography to give **3c** in 75% yield.

2.3 Preparation of 2-methyl-2-phenyltetrahydrofuran (3a) in water. Typical procedure.

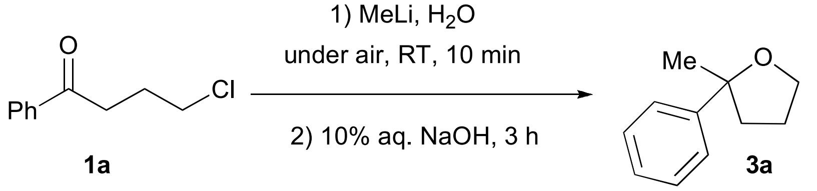

To a suspension of the ketone **1a** (0.5 mmol) in 1 mL of water, 0.94 mL of the commercially available MeLi (1.5 mmol in 1.6 M Et_2O solution), handled under argon using conventional Schlenk techniques, were quickly spread out at RT, under air, and vigorous stirring. After 10 min, 10 mL of 10% aq. NaOH were added and the mixture was stirred for additional 3 h, and then extracted with Et_2O (3 × 10 mL). The combined organic phases were dried over anhydrous Na_2SO_4 and the solvent was concentrated *in vacuo*. The crude product was purified by flash-chromatography to give **3a** in 75% yield.

3. Characterization Data

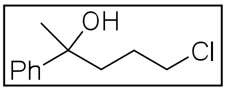

5-Chloro-2-phenylpentan-2-ol (2a): colourless oil. ^{1}H NMR (400 MHz, $CDCl_3$): δ 1.51 (s, 3 H); 1.54−1.61 (m, 1 H), 1.65−1.77 (m, 1 H), 1.81−1.92 (m, 2 H), 3.36–3.44 (m, 2 H); 7.15−7.19 (m, 1 H), 7.25−7.29 (m, 2 H), 7.34−7.36 (m, 2 H); ^{13}C NMR (100 MHz, $CDCl_3$): δ 27.4, 30.6, 41.4, 45.5, 74.4, 124.7, 126.8, 128.3, 147.3; FT-IR (film, cm^{-1}): 3436, 3058, 2956, 2925, 1601, 1445, 1028, 760, 700; GC-MS (70 eV) *m/z* (%): 198 (M^+, 5), 183 (9), 147 (11), 121 (100), 105 (19), 77 (10), 43 (15). Anal. Calcd. for $C_{11}H_{15}ClO$: C, 66.49; H, 7.61; Found: C, 66.84; H, 7.69.

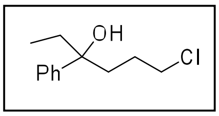

6-Chloro-3-phenylhexan-3-ol (2c): colourless oil. ^{1}H NMR (400 MHz, $CDCl_3$): δ 0.76 (t, J = 7.4 Hz, 3 H); 1.49−1.59 (m, 1 H), 1.77−2.01 (m, 5 H), 3.46−3.50 (m, 2 H); 7.23−7.26 (m, 1 H), 7.33−7.40 (m, 4 H); ^{13}C NMR (125 MHz, $CDCl_3$): δ 7.6, 27.0, 35.7, 39.9, 45.7, 76.9, 125.3, 126.5, 128.2, 145.2; FT-IR (film, cm^{-1}): 3467, 3027, 2963, 2929, 1602, 1446, 1310, 760, 701; GC-MS (70 eV) *m/z* (%): 212 (M^+, 2), 194 (6) 183 (75), 147 (61), 135 (55), 105 (100), 77 (10). HRMS calcd. for $C_{12}H_{17}ClO$ $(M + Na)^+$: 235.0866. Found: 235.0860.

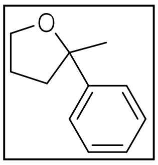

2-Methyl-2-phenyltetrahydrofuran (3a):[1] colourless oil. ^{1}H NMR (400 MHz, $CDCl_3$): δ 1.47−1.53 (m, 2 H), 1.57 (s, 3 H), 1.80−2.05 (m, 2 H), 3.55−3.65 (m, 2 H), 7.21−7.44 (m, 5 H); ^{13}C NMR (100 MHz, $CDCl_3$): δ 26.9, 30.2, 40.9, 62.5, 74.2, 124.9, 125.8, 125.9, 126.3, 127.2, 127.2, 128.0, 128.2, 128.2, 144.7, 144.8, 148.0; FT-IR (film, cm^{-1}): 3016, 2972, 2927, 2869, 1492, 1445, 1098, 1068, 1037, 763, 701; ESI-MS: 163.2 (M^+ + 1).

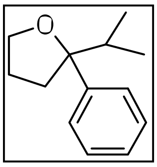

2-Isopropyl-2-phenyltetrahydrofuran (3b):[2] colourless oil. ^{1}H NMR (500 MHz, $CDCl_3$): δ 0.82 (d, J=6.5 Hz, 3 H), 0.87 (d, J=6.5 Hz, 3 H), 1.70−1.73 (m, 1 H), 1.90−1.93 (m, 1 H), 2.00−2.04 (m, 1 H), 2.06−2.10 (m, 1 H), 2.22−2.26 (m, 1 H),

3.76−3.82 (m, 1 H), 3.93−3.97 (m, 1 H), 7.21−7.38 (m, 5 H); ^{13}C NMR (125 MHz, $CDCl_3$): δ 17.5, 18.5, 25.7, 35.5, 37.9, 67.1, 89.5, 126.2, 126.3, 127.5, 145.0; FT-IR (film, cm^{-1}): 2965, 2873, 1489, 1469, 1445, 1382, 1364, 1055, 760, 703; ESI-MS: 189.1 (M^{-} − 1).

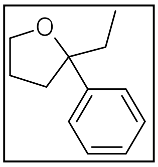

2-Ethyl-2-phenyltetrahydrofuran (3c):[3] colourless oil. ^{1}H NMR (400 MHz, $CDCl_3$): δ 0.76 (t, *J* = 7.4 Hz, 3 H), 1.86−1.74 (m, 3 H), 1.98−1.90 (m, 1 H), 2.07−2.00 (m, 1 H), 3.86−3.90 (m, 1 H), 3.94−4.00 (m, 1 H), 7.19−7.23 (m, 1 H), 7.37−7.29 (m, 4 H); ^{13}C NMR (100 MHz, $CDCl_3$) δ: 8.7, 25.6, 35.0, 37.7, 67.4, 77.4, 87.1, 125.3, 127.8, 146.5; FT-IR (film, cm^{-1}): 3024, 2967, 2929, 1492, 1446, 1057, 758, 701; GC-MS (70 eV) *m/z* (%): 176 (M^{+}, 3), 147 (100), 105 (55), 77 (16).

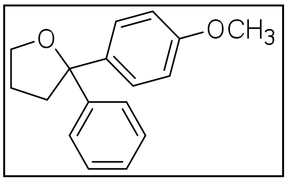

2-(4-Methoxyphenyl)-2-phenyltetrahydrofuran (3d):[4] colourless oil: ^{1}H NMR (400 MHz, $CDCl_3$): δ 1.91−1.98 (m, 2 H), 2.50−2.57 (m, 2 H), 3.76 (s, 3 H), 4.02−4.06 (m, 2 H), 6.81−6.84 (m, 2 H), 7.16−7.20 (m, 1 H), 7.27−7.36 (m, 4 H), 7.41−7.42 (m, 2 H); ^{13}C NMR (150 MHz, $CDCl_3$) δ: 25.5, 38.7, 55.5, 67.3, 87.8, 113.5, 125.8, 126.6, 127.1, 128.1, 138.5, 146.7, 158.4; FT-IR (film, cm^{-1}): 3059, 2953, 2876, 1610, 1509, 1250, 1055, 829; GC-MS (70eV), *m/z* (%): 254 (M^{+}, 25), 223 (10), 177 (100), 135 (50).

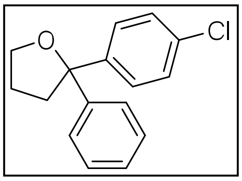

2-(4-Chlorophenyl)-2-phenyltetrahydrofuran (3e):[4] colourless oil. ^{1}H NMR (600 MHz, $CDCl_3$): δ 1.95−2.03 (m, 1 H), 2.07−2.14 (m, 1 H), 2.55−2.60 (m, 1 H), 3.03−3.07 (m, 1 H), 3.96−4.05 (m, 2 H), 7.20−7.23 (m, 2 H), 7.27−7.33 (m, 6 H), 7.92−7.93 (m, 1 H); ^{13}C NMR (150 MHz, $CDCl_3$): δ 26.6, 36.3, 67.6, 87.6, 126.5, 126.8, 127.1, 127.6, 127.9, 128.4, 131.0, 132.0, 143.6, 143.8; FT-IR (film, cm^{-1}): 2918, 1492, 1459, 758, 697; GC-MS (70 eV) *m/z* (%): 258 (M^{+}, 22), 223 (28), 181 (80), 147 (100), 105 (57).

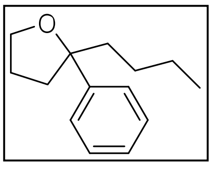

2-Butyl-2-phenyltetrahydrofuran (3f):[5] colourless oil. ^{1}H NMR (400 MHz, $CDCl_3$): δ 0.81 (t, *J* = 7.2 Hz, 3 H), 0.92–1.08 (m, 1 H), 1.18–1.29 (m, 3 H),

1.73–2.18 (m, 6 H), 3.85–3.97 (m, 2 H), 7.20–7.37 (m, 5 H); ^{13}C NMR (100 MHz, $CDCl_3$): δ 14.0, 23.1, 25.5, 26.6, 30.3, 67.4, 86.8, 125.2, 126.1, 127.9, 146.9; FT-IR (film, cm^{-1}): 2957, 2933, 2871, 1458, 1446, 1118, 1048, 763, 702; ESI-MS: 205 (M^{+} + 1).

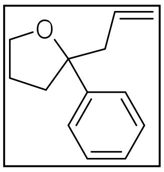

2-Allyl-2-phenyltetrahydrofuran (3g):[3] colourless oil. ^{1}H NMR (400 MHz, $CDCl_3$): δ 1.66–1.77 (m, 1 H), 1.85–1.92 (m, 1 H), 2.04–2.10 (m, 2 H), 2.43–2.48 (m, 1 H), 2.52–2.56 (m, 1 H), 3.81–3.85 (m, 1 H), 3.90–3.95 (m, 1 H), 4.91–4.96 (m, 2 H), 5.58–5.67 (m, 1 H), 7.12–7.17 (m, 1 H), 7.23–7.34 (m, 4 H); ^{13}C NMR (100 MHz, $CDCl_3$): δ 25.5, 37.2, 46.9, 67.7, 86.2, 117.4, 125.2, 126.3, 127.9, 134.3, 146.6; FT-IR (film, cm^{-1}): 2976, 2872, 1446, 1055, 914, 762, 703; GC-MS (70 eV) *m/z* (%): 188 (M+, 2), 147 (100), 105 (70), 77 (21).

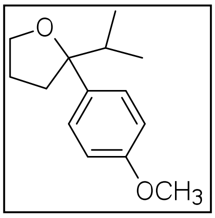

2-Isopropyl-2-(4-methoxyphenyl)tetrahydrofuran (3h): colourless oil. ^{1}H NMR (400 MHz, $CDCl_3$): δ 0.81 (d, *J* = 6.5 Hz, 3 H), 0.82 (d, *J* = 6.5 Hz, 3 H), 1.66–1.72 (m, 1 H), 1.85–1.91 (m, 1 H), 1.93–2.05 (m, 2 H), 2.19–2.24 (m, 1 H), 3.72–3.77 (m, 1 H), 3.80 (s, 3 H), 3.89–3.94 (m, 1 H), 6.83–6.86 (m, 2 H), 7.25–7.28 (m, 2 H); ^{13}C NMR (100 MHz, $CDCl_3$): δ 17.6, 18.5, 25.6, 35.3, 38.1, 55.2, 67.0, 89.3, 112.9, 127.5, 136.7, 158.1; FT-IR (film, cm^{-1}): 2962, 2874, 1610, 1508, 1464, 1294, 1246, 1176, 1058, 1039, 826, 799; GC-MS (70 eV) *m/z* (%): 220 (M^{+}, 13), 177 (100), 135 (30), 92 (23), 77 (14). Anal.Calcd. for $C_{14}H_{20}O_2$: C, 76.33; H, 9.15; Found: C, 76.40; H, 9.28.

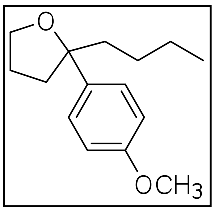

2-Butyl-2-(4-methoxyphenyl)tetrahydrofuran (3i): colourless oil. ^{1}H NMR (400 MHz, $CDCl_3$): δ 0.79 (t, *J* = 6.9 Hz, 3 H), 1.14–1.30 (m, 4 H), 1.67–1.79 (m, 3 H), 1.84–1.99 (m, 2 H), 2.10–2.16 (m, 1 H), 3.77 (s, 3 H), 3.80–3.85 (m, 1 H), 3.89–3.95 (m, 1 H), 6.81–6.84 (m, 2 H), 7.22–7.25 (m, 2 H); ^{13}C NMR (100 MHz, $CDCl_3$): δ 14.0, 23.1, 25.5, 26.7, 38.1, 42.4, 55.2, 67.2, 86.5, 113.2, 126.3, 138.8, 157.9; FT-IR (film, cm^{-1}): 2932, 1611, 1509, 1463, 1298, 1246, 1175, 1039, 830; GC-MS (70 eV) *m/z* (%): 234 (M^{+}, 15), 177 (100), 135 (43), 128 (12), 92 (14), 77 (5). Anal. Calcd. for $C_{15}H_{22}O_2$: C, 76.88; H, 9.46; Found: C, 77.11; H, 9.69.

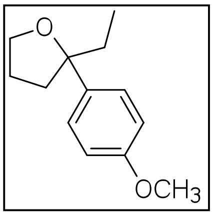

2-Ethyl-2-(4-methoxyphenyl)tetrahydrofuran (3j):[6] colourless oil. ^{1}H NMR (500 MHz, $CDCl_3$): δ 0.77 (t, J = 7.5 Hz, 3 H), 1.76–1.84 (m, 3 H), 1.92–2.05 (m, 2 H), 2.13–2.18 (m, 1 H), 3.81 (s, 3 H), 3.86–3.90 (m, 1 H), 3.94–3.98 (m, 1 H), 6.87 (d, J = 8.5 Hz, 2 H), 7.28 (d, J = 8.5 Hz, 2 H); ^{13}C NMR (125 MHz, $CDCl_3$): δ 9.1, 25.8, 35.4, 37.9, 55.5, 67.6, 87.2, 113.5, 126.7, 138.8, 158.2; FT-IR (film, cm^{-1}): 2966, 2934, 2875, 1610, 1582, 1510, 1442, 1299, 1176, 1037, 911, 830; ESI-MS: 207 (M^+ + 1).

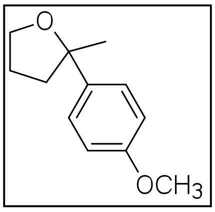

2-(4-Methoxyphenyl)-2-methyltetrahydrofuran (3k):[7] colourless oil. ^{1}H NMR (400 MHz, $CDCl_3$): δ 1.42 (s, 3 H), 1.87–1.93 (m, 1 H), 2.05–2.12 (m, 1 H), 3.80 (s, 3 H), 3.81–3.84 (m, 1 H) 3.89–3.94 (m, 1 H), 6.77–6.80 (m, 2H), 7.22–7.25 (m, 2H); ^{13}C NMR (100 MHz, $CDCl_3$): δ 27.2, 30.4, 41.1, 55.3, 67.1, 87.1, 113.3, 126.8, 140.3, 158.8; FT-IR (film, cm^{-1}): 2969, 2877, 1609, 1512, 1444, 1297, 1176, 1036, 910, 830; GC-MS (70 eV) *m/z* (%): 193 (M^+ + 1, 13), 177 (100), 175 (13), 151 (32), 135 (84) 77 (20).

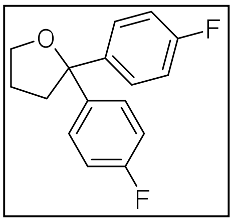

2,2-Bis(4-fluorophenyl)tetrahydrofuran (3l):[4] colourless oil. ^{1}H NMR (600 MHz; $CDCl_3$): δ 1.93−2.00 (m, 2 H), 2.50−2.54 (m, 2 H), 4.03−4.06 (m, 2 H), 6.96−7.01 (m, 4 H), 7.36−7.40 (m, 4 H); ^{13}C NMR (150 MHz, $CDCl_3$): δ 25.4, 38.8, 67.4, 87.2, 114.9 (d, $^2J_{C\text{-}F}$ = 21.2 Hz), 127.4 (d, $^3J_{C\text{-}F}$ = 8.0 Hz), 141.9 (d, $^4J_{C\text{-}F}$ = 3.3 Hz), 161.6 (d, $^1J_{C\text{-}F}$ = 240 Hz); FT-IR (film, cm^{-1}): 3055, 2959, 1598, 1506, 1266, 743; GC-MS (70eV), m/z (%): 260 (3), 219 (100), 123 (60). Anal. Calcd. for $C_{16}H_{14}F_2O$: C, 73.83; H, 5.42. Found: C, 74.02; H, 5.55.

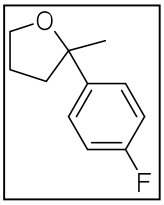

2-(4-Fluorophenyl)-2-methyltetrahydrofuran (3m): colourless oil. ^{1}H NMR (400 MHz, $CDCl_3$): δ 1.47 (s, 3 H), 1.72−1.80 (m, 1 H), 1.91−2.01 (m, 2 H), 2.08−2.15 (m, 1 H), 3.83−3.89 (m, 1 H), 3.94−3.99 (m, 1 H), 6.93−6.98 (m, 2 H), 7.08−7.13 (m, 1 H), 7.30−7.33 (m, 1 H); ^{13}C NMR (100 MHz, $CDCl_3$) δ: 17.0, 25.7, 39.5, 67.5, 83.9, 115.5 (d, $^2J_{C\text{-}F}$ = 21.4 Hz), 126.2 (d, $^3J_{C\text{-}F}$ = 7.5 Hz), 141.8, 161.3 (d, $^1J_{C\text{-}F}$ = 243.0 Hz); FT-IR (film, cm^{-1}): 3055, 2959, 1600, 1590, 1266, 1176, 912, 831; GC-MS (70 eV) *m/z* (%): 180 (M^+,6), 165 (100), 135 (7), 123 (77). Anal. Calcd. for $C_{11}H_{13}FO$: C, 73.31; H, 7.27. Found: C, 73.58; H, 7.51.

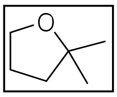

2,2-Dimethyltetrahydrofuran (3n):[8] colourless oil. ^{1}H NMR (400 MHz, $CDCl_3$): δ 1.24 (s, 6 H), 1.71 (t, *J* = 7.7 Hz, 2 H), 1.91–2.00 (m, 2 H), 3.84 (t, *J* = 6.9 Hz, 2 H); ^{13}C NMR (100 MHz, $CDCl_3$): δ 25.9, 27.7, 38.1, 67.1, 81.4; FT-IR (film, cm^{-1}): 2962, 1467, 1140, 609; ESI-MS: 100 (M^+).

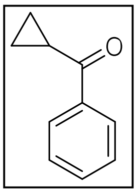

Cyclopropyl(phenyl)methanone (4a):[9] colourless oil. ^{1}H NMR (400 MHz, $CDCl_3$): δ 1.02 (ddd, *J* = 8.0, 7.0, 3.5 Hz, 2 H), 1.22 (ddd, *J* = 7.0, 4.5, 3.5 Hz, 2 H), 2.60–2.70 (m, 1 H), 7.43–7.47 (m, 2 H), 7.49–7.55 (m, 1 H), 7.96–8.02 (m, 2 H); ^{13}C NMR (100 MHz, $CDCl_3$): δ 11.6, 17.1, 127.9, 128.3, 132.7, 138.0, 200.5; FT-IR (film, cm^{-1}): 3031, 2960, 1668, 1597, 1579, 752, 700; ESI-MS: 146 (M^+).

^{1}H and ^{13}C NMR spectra of 2a

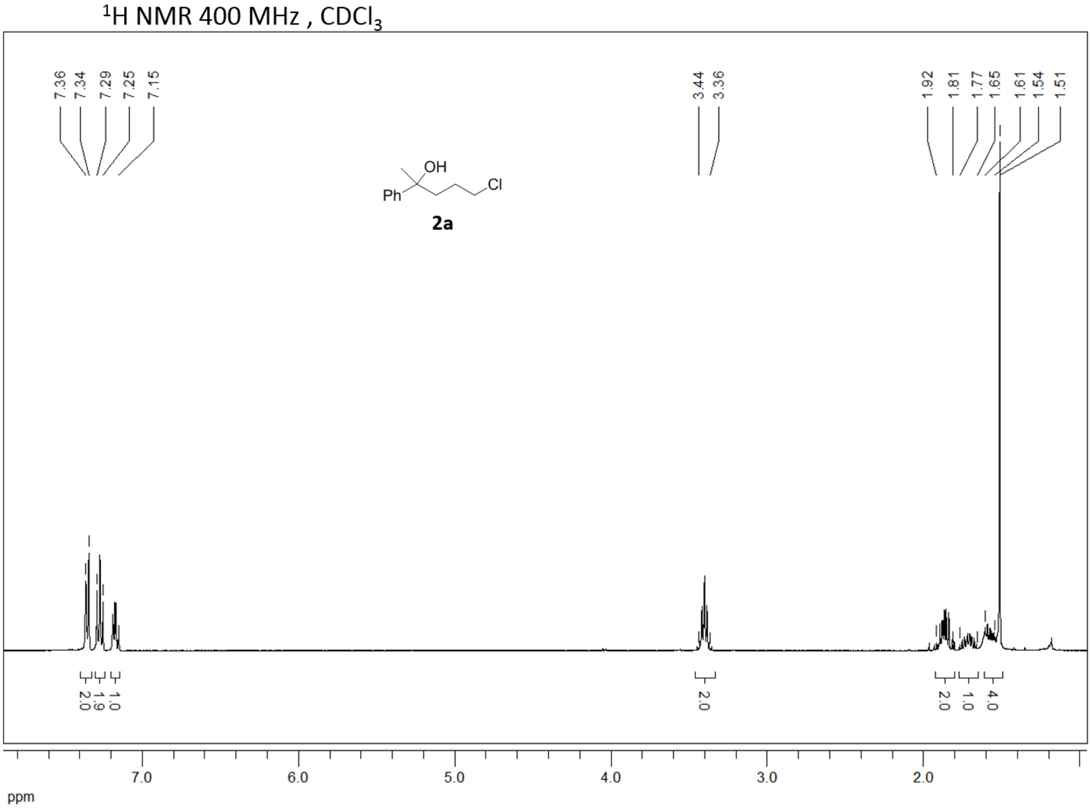

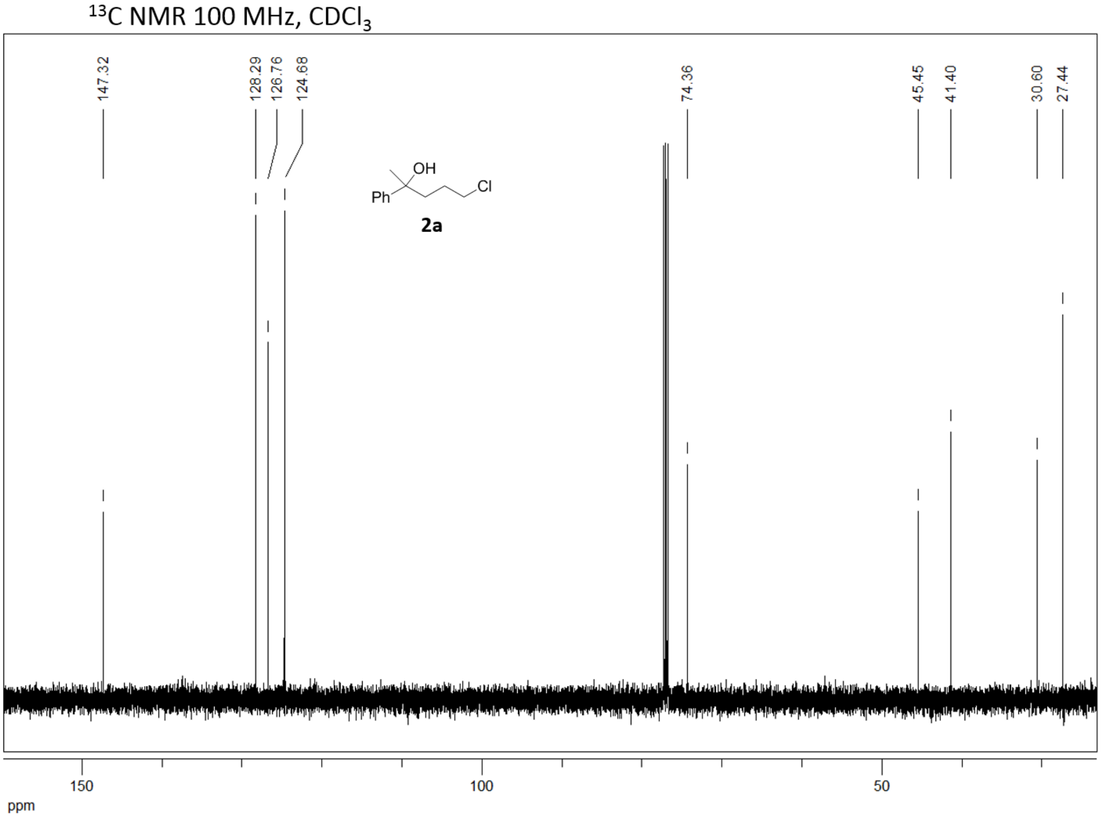

^{1}H and ^{13}C NMR spectra of 2c

^{1}H NMR 400 MHz , $CDCl_3$

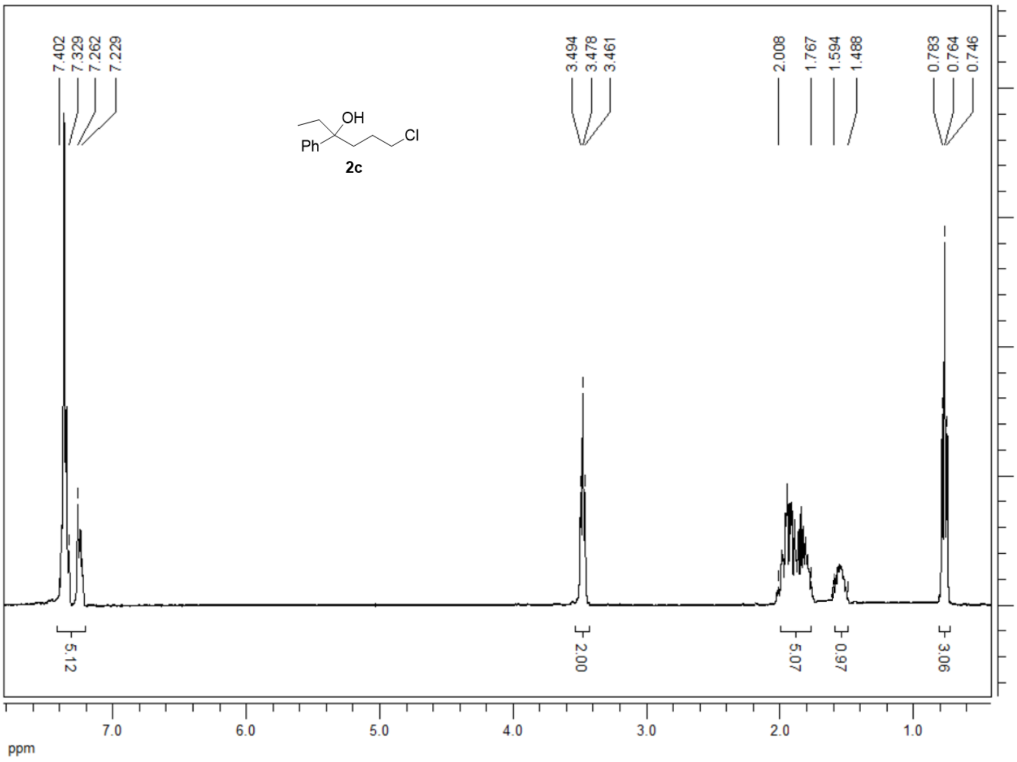

^{13}C NMR 125 MHz, $CDCl_3$

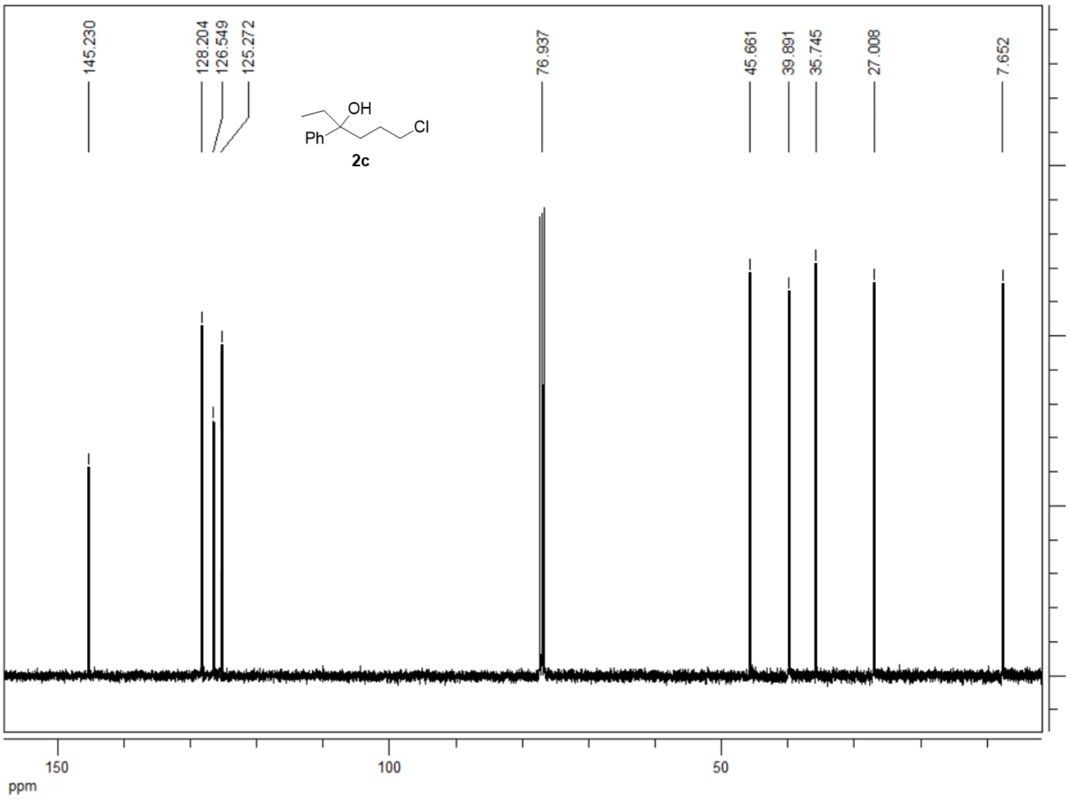

^{1}H and ^{13}C NMR spectra of 3h

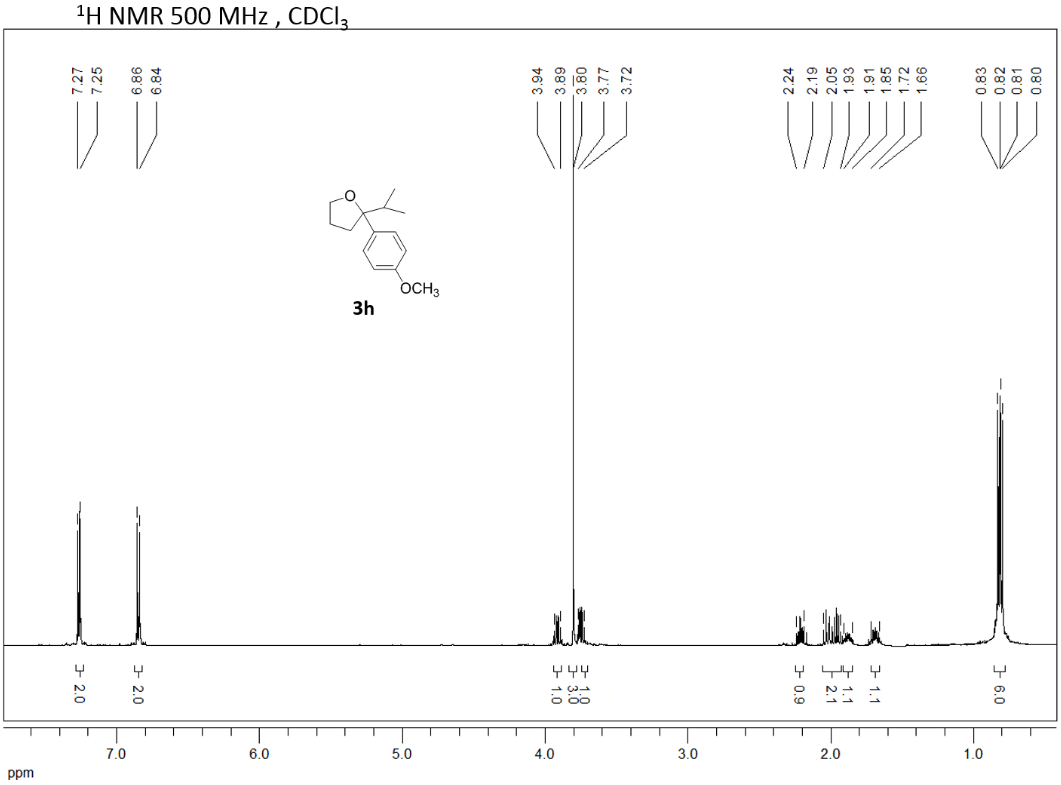

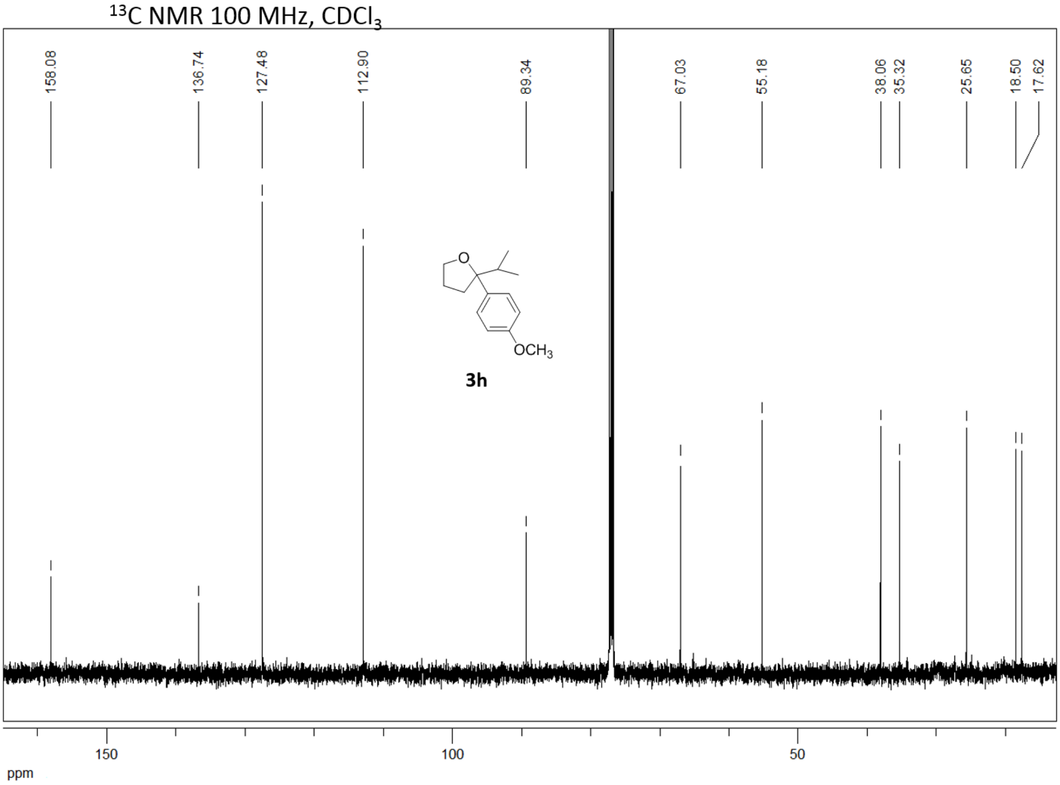

^{1}H and ^{13}C NMR spectra of 3i

^{1}H NMR 400 MHz , $CDCl_3$

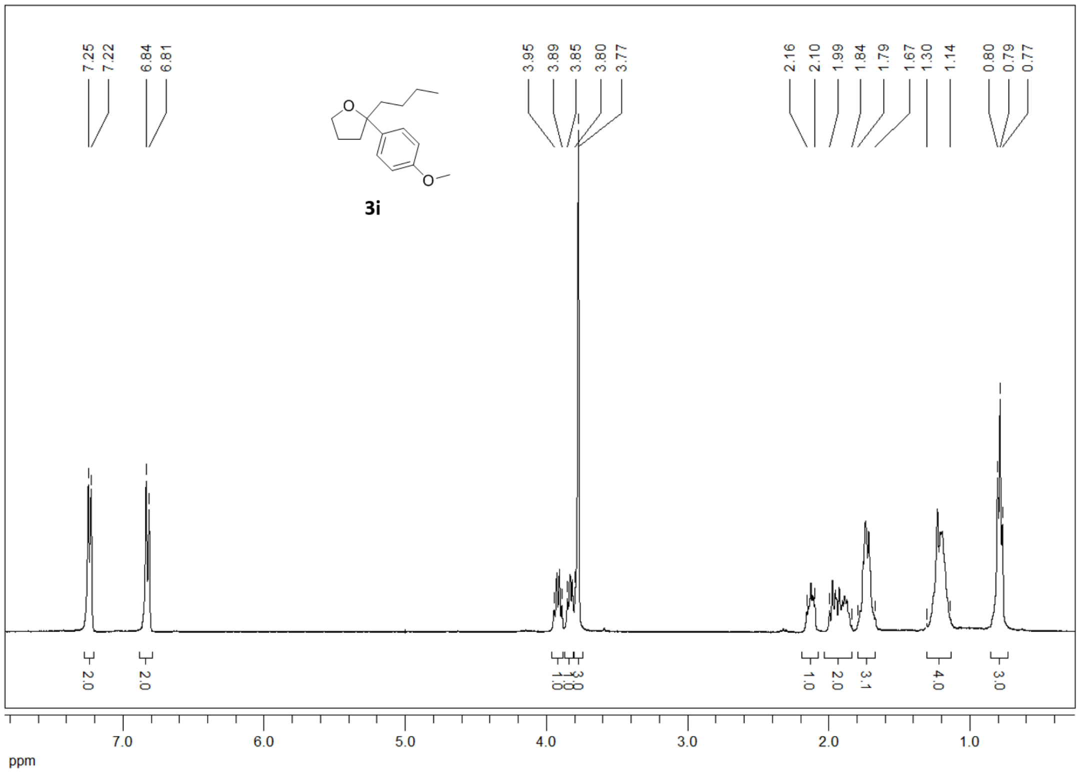

^{13}C NMR 100 MHz, $CDCl_3$

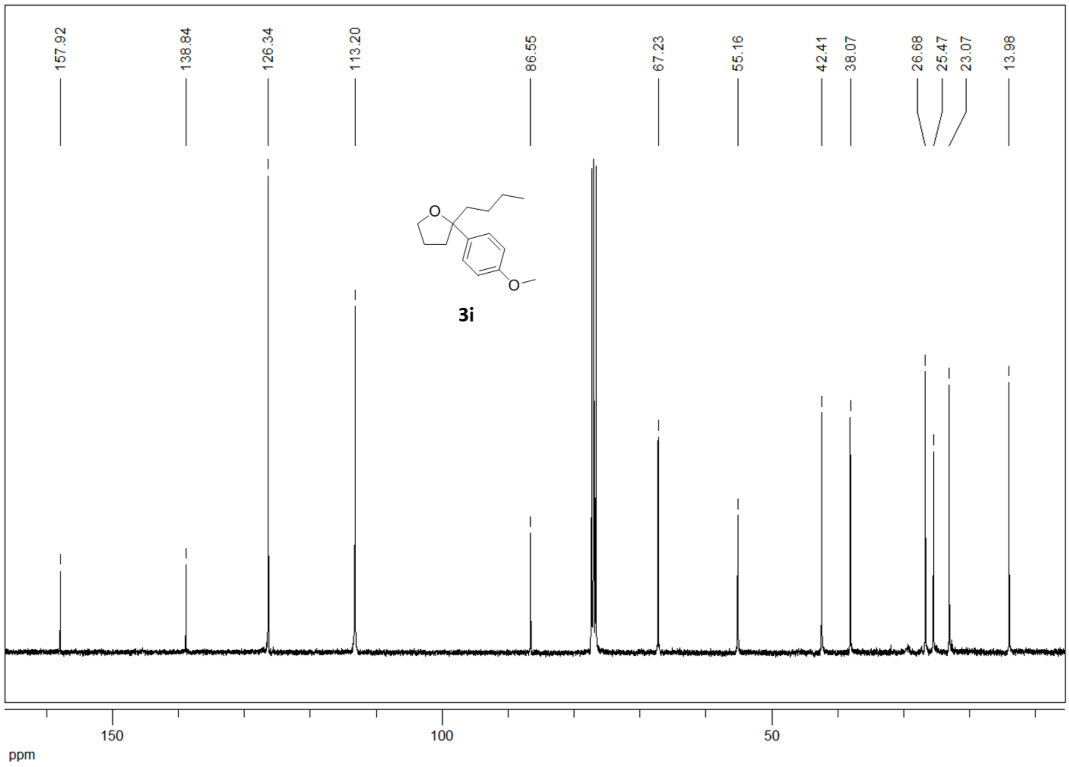

^{1}H and ^{13}C NMR spectra of 3m

^{1}H NMR 400 MHz , $CDCl_3$

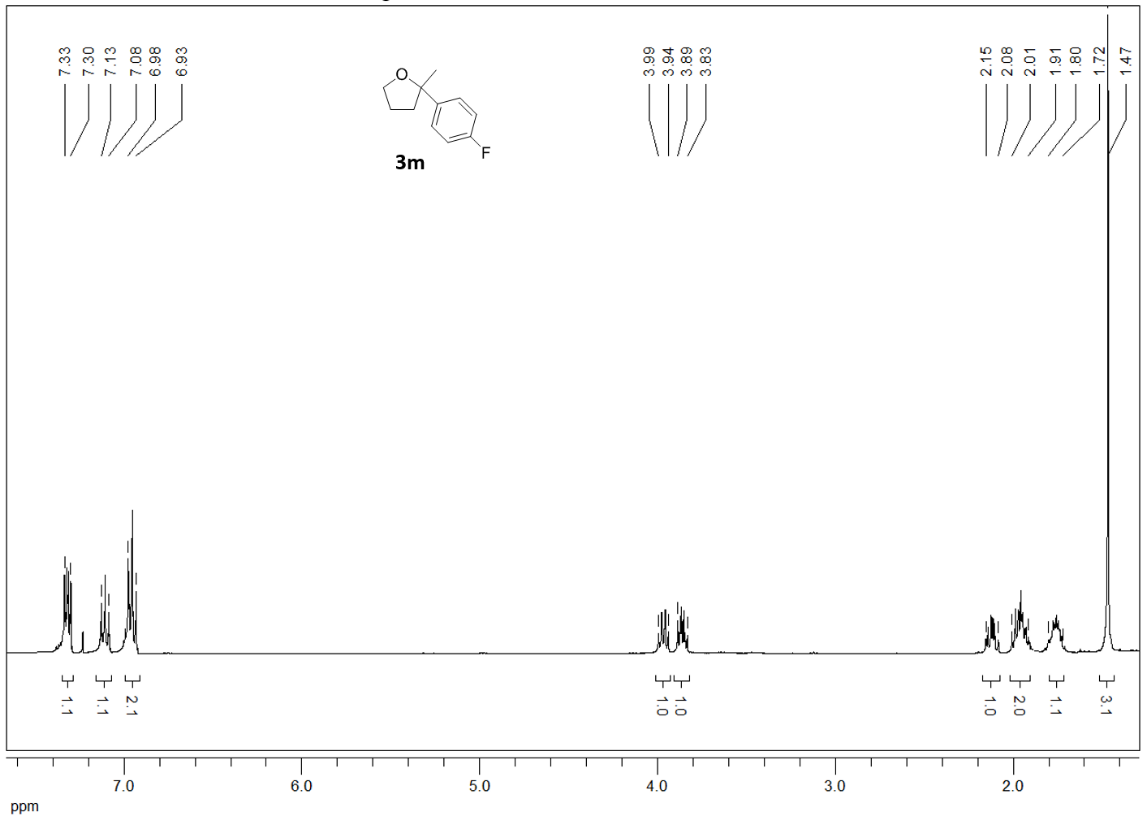

^{13}C NMR 100 MHz, $CDCl_3$

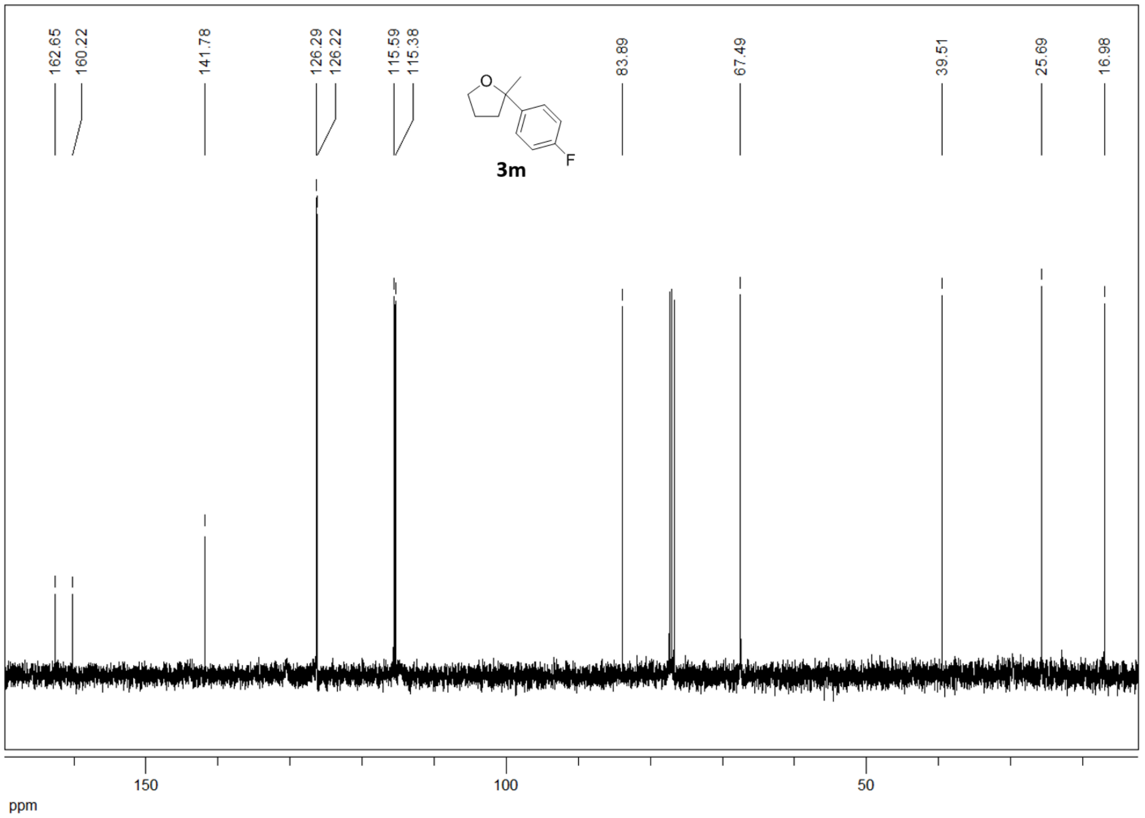

Supplement: Supplementary file 1 [file SC-007-C5SC03436A-s001.pdf]
